# Albumin-Bound Paclitaxel: Worthy of Further Study in Sarcomas

**DOI:** 10.3389/fonc.2022.815900

**Published:** 2022-02-10

**Authors:** Zhichao Tian, Weitao Yao

**Affiliations:** Department of Orthopedics, The Affiliated Cancer Hospital of Zhengzhou University and Henan Cancer Hospital, Zhengzhou, China

**Keywords:** albumin-bound paclitaxel, taxanes, sarcoma, breast cancer, lung cancer, pancreatic cancer

## Abstract

Taxanes (paclitaxel and docetaxel) play an important role in the treatment of advanced sarcomas. Albumin-bound paclitaxel (nab-paclitaxel) is a new kind of taxane and has many advantages compared with paclitaxel and docetaxel. Nab-paclitaxel is currently approved for the treatment of advanced breast, non-small cell lung, and pancreatic cancers. However, the efficacy of nab-paclitaxel in sarcomas has not been reviewed. In this review, we first compare the similarities and differences among nab-paclitaxel, paclitaxel, and docetaxel and then summarize the efficacy of nab-paclitaxel against various non-sarcoma malignancies based on clinical trials with reported results. The efficacy and clinical research progress on nab-paclitaxel in sarcomas are also summarized. This review will serve as a good reference for the application of nab-paclitaxel in clinical sarcoma treatment studies and the design of clinical trials.

## Introduction

Sarcomas (sarcomas in this review refer only to bone and soft tissue sarcomas, excluding gastrointestinal stromal tumors and Kaposi sarcoma) are malignant tumors derived from mesenchymal tissue (1). Sarcomas have low incidence, accounting for only about 2% of all newly diagnosed malignancies in humans each year (1, 2), yielding approximately 200,000 new cases (3, 4). A small number of these patients have metastases at initial diagnosis. Approximately 50% of newly diagnosed nonmetastatic cases eventually progress to an advanced stage (2, 4–6). The systemic treatment for advanced sarcomas is the same as that for other malignancies, including chemotherapy, targeted therapy, and immunotherapy (1, 7).

As chemotherapy drugs, paclitaxel and docetaxel play an important role in the treatment of advanced sarcomas (7–12). Paclitaxel and docetaxel are taxanes (13). Taxanes represent an important class of antineoplastic agents that interfere with microtubule function, leading to altered mitosis and cellular death *in vitro* (14); *in vivo*, intratumoral concentrations of taxanes cause cell death due to chromosome missaggregation in multipolar spindles (15). Paclitaxel was originally extracted from the Pacific yew tree, a small slow-growing evergreen, coniferous tree (16). Owing to the initial scarcity of paclitaxel, docetaxel, a semisynthetic analog of paclitaxel produced from the needles of the European yew tree, was developed (17, 18). Docetaxel differs from paclitaxel in its chemical structure in two positions (19). These small alterations make docetaxel different from paclitaxel in terms of water solubility, cellular effects, pharmacology, and other aspects (19–22) (**Table 1**).

**Table 1:** Comparison of nanoparticle albumin-bound paclitaxel (nab-paclitaxel) with solvent-based paclitaxel and docetaxel.

	Nab-paclitaxel	Docetaxel	Solvent-based paclitaxel	Reference
Launch date	2005	1996	1992	
Structure	Six or seven paclitaxel molecules bound non-covalently to an albumin molecule.	Differs from paclitaxel in two positions in its chemical structure.	–	(23, 24)
Solubilizer	Nanoparticle albumin	Polysorbate	Cremophor EL	(25)
The need for a special infusion tube	No	Yes	Yes	(25)
Infusion time (min)	20-30	60	120–180	
Corticosteroid premedication	No	Yes	Yes	
Indications	Breast cancer, lung cancer, and pancreatic cancer.	Breast cancer, lung cancer, prostate cancer, gastric cancer, and head/neck cancer.	Ovarian cancer, breast cancer, AIDS-related Kaposi sarcoma, lung cancer, and esophageal cancer.	
Differences in patients of different ages	No differences were noted for pharmacodynamic variables (grade 3 toxicity, dose reductions, or dose omissions) based on age. It is well-tolerated across all age groups.	Showing lower docetaxel clearance in elderly patients than in younger patients.	Older patients are more prone to develop paclitaxel-induced toxicity than their younger peers.	(26–28)
Preclinical studies	Compared with Cremophor EL-paclitaxel, nab-paclitaxel was associated with faster and deeper tissue penetration, shorter duration of high systemic exposure, and slower elimination of paclitaxel.	Docetaxel exhibited more potent cytotoxicity against different tumors than paclitaxel both *in vitro* and *in vivo*. As for tubulin promotion and blocking depolymerization, docetaxel, is about five-fold more potent than paclitaxel.	–	(29–31)
Prices	Significantly higher than docetaxel and solvent-based paclitaxel.	Significantly lower than nab-paclitaxel, similar with solvent-based paclitaxel.	–	(32–34)

Paclitaxel is currently one of the widely used antineoplastic agents with broad activity in several cancers, including breast cancer, ovarian cancer, endometrial cancer, squamous cell carcinoma, adenocarcinoma, non-small cell lung cancer (NSCLC), bladder cancer, cervical carcinoma, and esophageal cancer (35, 36). Paclitaxel is also used in the treatment of some sarcomas (8), especially angiosarcoma (11, 37). Docetaxel is a widely prescribed antineoplastic agent for a broad range of malignancies including breast cancer, lung cancer, head/neck cancer, prostate cancer, and gastric cancer (18, 21, 29, 38). The efficacy of docetaxel-based chemotherapy in the treatment of soft tissue sarcomas is comparable to that of first-line treatment with doxorubicin, but is considered inferior to doxorubicin chemotherapy because of its toxicity and administration complexity (39). Docetaxel-based chemotherapy is also considered a second-line treatment for advanced osteosarcoma (40). Nevertheless, paclitaxel and docetaxel have several clinical problems, including poor drug solubility and serious dose-limiting toxicities such as peripheral sensory neuropathy, myelosuppression, and allergic reactions. A number of these clinical problems have been associated with the solvents used for diluting these antineoplastic agents: Cremophor EL for paclitaxel and polysorbate 80 for docetaxel (23, 29, 41–43).

To overcome the clinical problems associated with the solvents used for diluting paclitaxel and docetaxel, albumin-bound paclitaxel (nab-paclitaxel) was developed (44). Nab-paclitaxel is an albumin-bound, 130-nm particle size formulation of paclitaxel that is devoid of any solvents or ethanol (14, 44). The lyophilized formulation, comprised of albumin and paclitaxel, is reconstituted in 0.9% NaCl to form a colloidal suspension (45). Compared with paclitaxel, despite having the same active ingredient (paclitaxel), the two different formulations exhibit unique efficacy and safety profiles (14, 30). The development of albumin-bound nanotechnology as a delivery system for paclitaxel neutralized its hydrophobicity and provided better pharmacokinetic and pharmacodynamic characteristics (45, 46). Nab-paclitaxel showed increased endothelial cell transport, intratumor paclitaxel concentrations, and antitumor activity compared with cremophor-based paclitaxel (31, 44) (**Table 1**). To date, nab-paclitaxel has been indicated for the treatment of metastatic breast cancer, locally advanced or metastatic NSCLC, and metastatic pancreatic cancer in the USA (47). It also showed promising efficacy in many other solid tumors (48–50). Currently, a number of clinical trials on the efficacy of nab-paclitaxel in various malignancies are being conducted (https://clinicaltrials.gov). The available evidence shows that the efficacy and safety of nab-paclitaxel are superior to those of paclitaxel and docetaxel in a variety of malignancies (22, 51–53). Studies have also confirmed the effectiveness of nab-paclitaxel in the treatment of sarcomas (54–57). However, it is not clear whether this formulation is more effective than conventional paclitaxel and docetaxel. In this review, we first considered the efficacy of nab-paclitaxel in the treatment of various non-sarcoma malignancies, and then focused on related studies of nab-paclitaxel in the treatment of sarcomas, with the aim of providing a reference to establish a clinical study design and to support clinical treatment of sarcomas.

## Efficacy of Nab-Paclitaxel in Non-Sarcoma Malignancies

To fully evaluate the efficacy of nab-paclitaxel in the treatment of malignancies, systematic hand and online searches were conducted. The inclusion criteria for literature and clinical trials were as follows: 1) published in 2011 and later; 2) prospective phase II or III clinical trials; 3) trials of nab-paclitaxel monotherapy or combination therapy of malignancies; 4) trials with a registration number; and 5) trials with complete data published in open-access journals. The retrieved clinical trials and literature were classified according to the histopathological type.

### Breast Cancer

Breast cancer was the first indication for which nab-paclitaxel was approved by the US Food and Drug Administration (FDA). The results of the first phase III trial in women with metastatic breast cancer showed that nab-paclitaxel demonstrated significantly higher response rates than standard paclitaxel (33% vs. 19%, respectively) and significantly longer progression-free survival (23.0 vs. 16.9 weeks, respectively) (58). This led to FDA approval of nab-paclitaxel in 2005 for second-line and above-line treatment of metastatic breast cancer. Since then, nab-paclitaxel has been widely studied in breast cancer, including attempts to use this formulation as a first-line treatment for metastatic breast cancer. However, these studies have not yielded sufficiently positive results to enable replacement of traditional first-line treatment (59–61). In a multicenter, randomized, comparative phase III study reported in 2018, atezolizumab plus nab-paclitaxel treatment prolonged progression-free survival among patients with metastatic triple-negative breast cancer in both the intention-to-treat population and the programmed death ligand 1-positive subgroup (62). This led to FDA approval of nab-paclitaxel in combination with atezolizumab for first-line treatment of metastatic breast cancer in 2019.

Several recent clinical trials have shown that nab-paclitaxel is effective for the treatment of non-metastatic breast cancer in the neoadjuvant setting. Nab-paclitaxel improved the pathological complete response rate and event-free survival compared with traditional taxanes, and with acceptable toxicities (63). Current studies of nab-paclitaxel in breast cancer have focused on dose adjustment and combination regimens with other drugs (64–68). In conclusion, the application of nab-paclitaxel in breast cancer has gradually improved from second-line and above- to first-line and neoadjuvant therapy. The application regimen has gradually evolved from a single drug to combinations with other chemotherapy drugs and/or with immunotherapy. Nab-paclitaxel is now a basic drug in the treatment of breast cancer. With the invention and application of new anti-tumor drugs, the combination of nab-paclitaxel with other drugs, and different application scenarios in the treatment of breast cancer, the role of nab-paclitaxel will be further studied.

### Lung Cancer

The results of a multicenter, randomized, controlled phase III clinical trial showed that nab-paclitaxel demonstrated a significantly higher objective response rate than solvent-based paclitaxel (33% vs. 25%) as first-line therapy in patients with advanced NSCLC (43). This led to FDA approval of nab-paclitaxel in combination with carboplatin as first-line treatment for advanced NSCLC in 2012. Since then, nab-paclitaxel has been widely studied in lung cancer. Subsequent clinical trials demonstrated the good activity of nab-paclitaxel combined with carboplatin in different patients with advanced NSCLC (69–72); however, nab-paclitaxel-based therapy had unsatisfactory efficacy in advanced small cell lung cancer (73, 74). Other clinical trials have shown that nab-paclitaxel-based chemotherapy worked as well as, or better than, other conventional drugs and regimens in advanced NSCLC (75–78). Recent clinical trial results show that nab-paclitaxel-based chemoradiotherapy has promising efficacy in the treatment of locally advanced NSCLC (79–81). Furthermore, the combination of nab-paclitaxel-based chemotherapy and immunotherapy can also achieve good efficacy (82–85). In conclusion, the clinical study of nab-paclitaxel in lung cancer has deepened our understanding of the treatment of advanced NSCLC patients who failed multi-line treatment by showing efficacy in multi-drug combination therapy, first-line, or neoadjuvant therapy in different subgroups. Clinical studies of nab-paclitaxel in lung cancer will become increasingly detailed and complex.

### Pancreatic Cancer

The results of an international, multicenter, open label, randomized phase III study showed that treating metastatic pancreatic cancer patients with gemcitabine plus nab-paclitaxel (GnP) as first-line therapy resulted in a significantly higher median overall survival time than treatment with gemcitabine alone (8.5 vs. 6.7 months) (86). This led to FDA approval of nab-paclitaxel in combination with gemcitabine for first-line treatment of metastatic pancreatic adenocarcinoma in 2013. Since then, nab-paclitaxel has been widely studied for treating pancreatic cancer. These clinical trials and studies can be grouped into four categories: 1. Continued testing of GnP in different groups of patients with advanced pancreatic cancer, such as elderly patients and patients with poor physical performance (87–89); 2. Comparing the efficacy and safety of GnP with FOLFIRINOX (a conventional chemotherapy regimen) for pancreatic cancer (90–93); 3. Testing new multidrug combination regimens based on nab-paclitaxel (94–97); and 4. Preoperative adjuvant therapy in patients with locally advanced pancreatic cancer (98–100). In conclusion, the research and application of nab-paclitaxel in pancreatic cancer are also a complex process from single to multi-drug combination therapy, from an ordinary to a special patient group, and from advanced first-line therapy to neoadjuvant therapy. According to various trial results, nab-paclitaxel has clear efficacy in the treatment of pancreatic cancer and has been considered a basic drug for adjuvant chemotherapy in this disease.

### Other Cancers

As the price of nab-paclitaxel has decreased, the numbers of studies and trials on this agent in various cancers have increased significantly. At present, clinical trial results have shown that nab-paclitaxel has promising efficacy in gastric cancer, melanoma, gynecological malignancy, urothelial carcinoma, head and neck squamous cell carcinoma, biliary tract carcinoma, and nasopharyngeal carcinoma (50, 52, 101–104). The research and application of nab-paclitaxel in every kind of malignant tumor have gradually evolved toward the direction of combined and perioperative treatment, similar with its path in breast cancer, lung cancer, and pancreatic cancer (105–108). It is believed that, with further research, the status of nab-paclitaxel in various malignant tumors will gradually be equal to or even exceed that of traditional taxanes.

## Efficacy of Traditional Taxanes and Nab-Paclitaxel in Sarcomas

### Efficacy of Traditional Taxanes in Sarcomas

Taxanes are one of the important class of drugs in the treatment of sarcomas and are recommended as systemic chemotherapy drugs in the National Comprehensive Cancer Network guidelines for the treatment of sarcomas (109, 110). Recent data demonstrate that paclitaxel causes cell death due to chromosome missegregation on multipolar spindles (15). As the first taxanes, paclitaxel was proved to be ineffective in most soft tissue sarcomas, except angiosarcoma, in the 1990s (111). At present, paclitaxel is the first choice of chemotherapy for vasogenic malignancies (112, 113). With the application of targeted drugs in the treatment of sarcoma, the combination of paclitaxel and targeted drugs also shows promising activity (114). However, because of the simultaneous use of steroids, paclitaxel is obviously not suitable for use in combination with immunotherapeutic drugs.

As described earlier in this review, docetaxel, an improved and invented drug based on paclitaxel, is as effective as or better than paclitaxel in a variety of malignancies (**Table 1**) (19). Therefore, researchers initially concluded that the drug should also be more effective in sarcoma (17). However, the results of initial clinical trial have shown that docetaxel monotherapy has limited activity in sarcoma (115). Fortunately, subsequent clinical trials have shown that gemcitabine in combination with docetaxel is more effective (116, 117). This has led to extensive research and application of docetaxel and gemcitabine combination therapy in sarcomas. Currently, gemcitabine plus docetaxel is considered to have similar efficacy to doxorubicin as first-line treatment in previously untreated advanced unresectable or metastatic soft-tissue sarcomas (39). It is important to note that docetaxel plus gemcitabine combined with pazopanib is too toxic to be used. This has limited the application of this approach in the era of targeted therapy (118). In addition, docetaxel is also clearly not suitable for use in combination with immunotherapy drugs, since steroid use is also required.

### Efficacy of Nab-Paclitaxel in Sarcomas

As a new and improved taxane chemotherapeutic drug, nab-paclitaxel has many advantages compared with paclitaxel and docetaxel (**Table 1**) (46). Like other drugs, the inhibitory effect of paclitaxel on tumor tissue is dose-dependent (15). Mechanistically, because it is less toxic and more penetrating, a higher tumor/plasma paclitaxel drug ratio in favor of nab-paclitaxel was observed (119). Because secreted protein acidic and rich in cysteine (SPARC) shows high binding affinity to albumin, a study has proved that pediatric sarcoma xenografts expressing SPARC would show enhanced uptake and accumulation of nab- paclitaxel (120). We therefore infer that nab-paclitaxel is likely to be more effective in sarcomas. However, as a new taxane drug, nab-paclitaxel has not been studied extensively in sarcomas. We systematically searched all relevant literature on the treatment of bone and soft tissue sarcomas with nab-paclitaxel, and the results are shown in **Table 2**. Current evidence shows that nab-paclitaxel has promising effects in the treatment of angiosarcoma, epithelioid sarcoma, leiomyosarcoma, and other subtypes of soft tissue sarcomas (**Table 2**). GnP showed mild activity in Ewing sarcoma (**Table 2**). However, the evidence level in these studies was not high. Compared with research on nab-paclitaxel in other malignant tumors and that on solvent-based paclitaxel in sarcomas, research on nab-paclitaxel in sarcomas is in its infancy.

**Table 2:** Studies on the treatment of sarcomas with nanoparticle albumin-bound paclitaxel (nab-paclitaxel).

Publication year	Research type	Research content	Conclusions	Reference
2014	Preclinical study	Preclinical evaluation of nab-paclitaxel in an Ewing sarcoma xenograft and an osteosarcoma model.	Observed growth inhibition and improved survival with nab-paclitaxel in an Ewing sarcoma xenograft; activity was additive with gemcitabine in an osteosarcoma model.	(121)
2016	Case report	Reported a patient with angiosarcoma of the thoracic wall who responded well to nab-paclitaxel.	After 4 courses of chemotherapy, the tumors regressed remarkably. Nab-paclitaxel may be an effective treatment option for recurrent or metastatic angiosarcoma.	(122)
2017	Case report	Reported a patient with cardiac angiosarcoma associated with disseminated intravascular coagulation who was successfully treated with nab-paclitaxel.	This report suggests the potential utility of nab-paclitaxel for angiosarcoma.	(123)
2018	Retrospective study	Reviewed the records of sixteen relapsed/refractory sarcoma patients who received gemcitabine/nab-paclitaxel.	Gemcitabine/nab-paclitaxel is a relatively safe regimen with mainly hematologic toxicities. It offers a well-tolerated, palliative option providing clinical benefit in a subset of patients.	(56)
2018	Phase I study	Sixty-four patients with recurrent/refractory extracranial solid tumours received nab-paclitaxel on days 1, 8, and 15 every 4 weeks at 120, 150, 180, 210, 240, or 270 mg/m^2^ to establish the maximum tolerated dose and recommended phase II dose.	Nab-paclitaxel 240 mg/m^2^ qw 3/4 (nearly double the adult recommended mono-therapy dose for this schedule in metastatic breast cancer) was selected as the recommended phase II dose based on the tolerability profile, pharmacokinetics, and antitumor activity.	(54)
2019	Preclinical study	Determined the efficacy of nab-paclitaxel in combination with gemcitabine, compared to conventional drugs, such as docetaxel, gemcitabine combined with docetaxel on an undifferentiated/unclassified soft-tissue sarcoma from a striated muscle implanted in the right biceps femoris muscle of nude mice to establish a patient-derived orthotopic xenograft model.	The results suggested that the combination of gemcitabine and nab-paclitaxel could be a promising therapeutic strategy for undifferentiated/unclassified soft-tissue sarcomas.	(57)
2020	Phase II study	Forty-two paediatric patients with recurrent or refractory Ewing sarcoma, neuroblastoma, or rhabdomyosarcoma received 240 mg/m^2^ nab-paclitaxel on days 1, 8, and 15 of each 28-day cycle. The primary end-point was the overall response rate.	In this phase II study, limited activity was observed; however, the safety of nab-paclitaxel in pediatric patients was confirmed.	(124)
2020	Phase II study	Using a Simon’s two-stage design to identify a response rate of ≥ 35%, eleven patients with relapsed Ewing sarcoma received nab-paclitaxel 125 mg/m^2^ followed by gemcitabine 1000 mg/m^2^ i.v. on days 1, 8, and 15 of four-week cycles. Immunohistochemical analysis of archival tissue was performed to identify possible biomarkers of response.	In patients with heavily pretreated Ewing sarcoma, the confirmed response rate of 9% was similar with multi-institutional studies of gemcitabine and docetaxel.	(125)
2020	Retrospective study	The clinical data of seventeen sarcoma patients who received nab-paclitaxel/gemcitabine chemotherapy between January 2019 and February 2020 were retrospectively analysed. All these patients were treated with nab- paclitaxel/gemcitabine only after doxorubicin-based chemotherapy had failed.	Nab-paclitaxel/gemcitabine combination chemotherapy is comparatively effective in the treatment of soft tissue sarcoma, demonstrates low toxicity, and is worthy of further study.	(126)
2021	Case report	Reported a pancreatic leiomyosarcoma patient who was successfully treated with nab-paclitaxel and gemcitabine.	Gemcitabine plus nab‐paclitaxel therapy might be the first choice for soft-tissue sarcoma and leiomyosarcoma.	(127)

## Discussion

In this review, we first determined the similarities and differences between nab-paclitaxel and other taxanes. We found that nab‐paclitaxel has many advantages over traditional solvent-based taxanes in terms of structure and performance. We then summarized the therapeutic efficacy and clinical trial process of nab-paclitaxel in non-sarcoma malignancies. The application of nab-paclitaxel in various malignant tumors is a simple, but complex, process. Finally, we reviewed the application of traditional taxanes and nab-paclitaxel in treating sarcomas. At present, research on nab-paclitaxel in sarcomas is in its infancy. Current evidence shows that nab-paclitaxel may have better activity than traditional taxanes in some subtypes, such as angiosarcoma, epithelioid sarcoma, and leiomyosarcoma.

**Figure 1:**
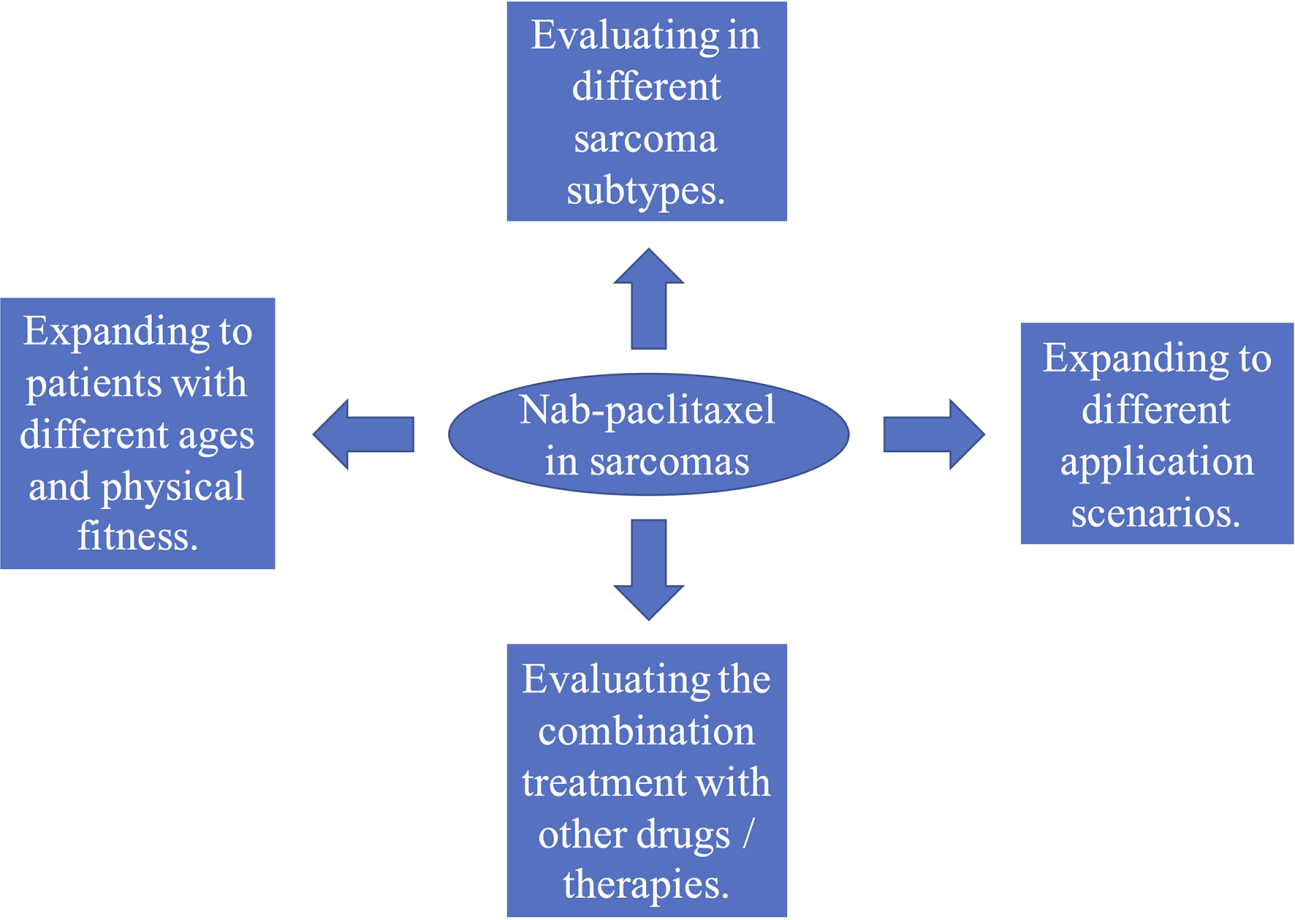
Four directions for clinical studies on albumin-bound paclitaxel (nab-paclitaxel) in sarcomas.

Based on the above review, we believe that the use of nab-paclitaxel in treating sarcomas is worthy of further study. These studies can be conducted in at least four different directions (**Figure 1**). 1) Sarcomas are divided into dozens of histological subtypes, and each subtype has a different sensitivity to taxanes. Therefore, the efficacy of nab-paclitaxel therapy should be evaluated for each subtype. 2) Combined application of nab-paclitaxel with other drugs (such as chemotherapy drugs, targeted drugs, and immunotherapy drugs), or radiotherapy and interventional therapy, should be studied further. For example, traditional taxanes cannot be effectively combined with immunotherapy because they require concurrent administration of steroids. However, studies have shown that nab‐paclitaxel can regulate the immune microenvironment and sensitize cancers to immunotherapy (128–130). This suggests that nab‐paclitaxel combined with immunotherapy for sarcomas may have a better curative effect. 3) Expanding the application of nab-paclitaxel to patients of different ages and patients with poor physical fitness should be explored. Compared with traditional taxanes, nab-paclitaxel has fewer toxic side effects and lower requirements for infusion conditions. Thus, it may have significant advantages in both young and elderly patients, or those with poor physical fitness. 4) Expand the application of nab-paclitaxel to different application scenarios of sarcoma treatment, such as neoadjuvant therapy and first-line treatment of advanced cancer patients.

Over the past decade, the main reason for restricting the wide use of nab-paclitaxel has been its high price. At present, nab-paclitaxel has successfully entered the list of essential drugs in China’s national medical insurance system, and its price has been greatly reduced. This indicates that the application and research of nab-paclitaxel in various malignant tumors, including sarcomas, will expand.

In conclusion, nab‐paclitaxel has been shown to have better activity than paclitaxel and docetaxel in many types of malignant tumors. Although research on its efficacy in sarcomas is in its infancy, it has been shown to have promising efficacy in some sarcoma subtypes. Overall, nab-paclitaxel is expected to become one of the important basic drugs in the field of sarcoma treatment.

## Author Contributions

All authors listed have made a substantial, direct, and intellectual contribution to the work, and approved it for publication.

## Conflict of Interest

The authors declare that the research was conducted in the absence of any commercial or financial relationships that could be construed as a potential conflict of interest.

## Publisher’s Note

All claims expressed in this article are solely those of the authors and do not necessarily represent those of their affiliated organizations, or those of the publisher, the editors and the reviewers. Any product that may be evaluated in this article, or claim that may be made by its manufacturer, is not guaranteed or endorsed by the publisher.
